# An Immune-Related Gene Pair Index Predicts Clinical Response and Survival Outcome of Immune Checkpoint Inhibitors in Melanoma

**DOI:** 10.3389/fimmu.2022.839901

**Published:** 2022-02-24

**Authors:** Junya Yan, Xiaowen Wu, Jiayi Yu, Yan Kong, Shundong Cang

**Affiliations:** ^1^ Department of Oncology, Henan Provincial People’s Hospital, Zhengzhou University People’s Hospital, Henan University People’s Hospital, Zhengzhou, China; ^2^ Key Laboratory of Carcinogenesis and Translational Research (Ministry of Education/Beijing), Department of Melanoma and Sarcoma, Peking University Cancer Hospital & Institute, Beijing, China; ^3^ Key Laboratory of Carcinogenesis and Translational Research (Ministry of Education/Beijing), Department of Radiation Oncology, Peking University Cancer Hospital & Institute, Beijing, China

**Keywords:** immune-related gene pair index (IRGPI), immune checkpoint inhibitor (ICI), melanoma, prediction, immune infiltration

## Abstract

The durable responses and favorable long-term outcomes are limited to a proportion of advanced melanoma patients treated with immune checkpoint inhibitors (ICI). Considering the critical role of antitumor immunity status in the regulation of ICI therapy responsiveness, we focused on the immune-related gene profiles and aimed to develop an individualized immune signature for predicting the benefit of ICI therapy. During the discovery phase, we integrated three published datasets of metastatic melanoma treated with anti-PD-1 (n = 120) and established an immune-related gene pair index (IRGPI) for patient classification. The IRGPI was constructed based on 31 immune-related gene pairs (IRGPs) consisting of 51 immune-related genes (IRGs). The ROC curve analysis was performed to evaluate the predictive accuracy of IRGPI with AUC = 0.854. Then, we retrospectively collected one anti-PD-1 therapy dataset of metastatic melanoma (n = 55) from Peking University Cancer Hospital (PUCH) and performed the whole-transcriptome RNA sequencing. Combined with another published dataset of metastatic melanoma received anti-CTLA-4 (VanAllen15; n = 42), we further validated the prediction accuracy of IRGPI for ICI therapy in two datasets (PUCH and VanAllen15) with AUCs of 0.737 and 0.767, respectively. Notably, the survival analyses revealed that higher IRGPI conferred poor survival outcomes in both the discovery and validation datasets. Moreover, correlation analyses of IRGPI with the immune cell infiltration and biological functions indicated that IRGPI may be an indicator of the immune status of the tumor microenvironment (TME). These findings demonstrated that IRGPI might serve as a novel marker for treating of melanoma with ICI, which needs to be validated in prospective clinical trials.

## Introduction

Malignant melanoma is an aggressive malignant tumor with a poor clinical prognosis, the incidence of which is increasing globally (1–3). With the development of immunotherapy, immune checkpoint inhibitors (ICI) therapy has been approved as the standard treatment for melanoma (4–8). According to the reports from multiple clinical trials (9–12), the overall response rate (ORR) of PD-1 blockade with nivolumab or pembrolizumab ranged from 26% to 44%, thus indicating almost 50% of patients with severely progressed melanoma do not obtain complete or partial response, with roughly 24% reach a stable disease only. Notably, as the main subtypes of Asian patients with melanoma, only 10~20% of acral and mucosal cases can benefit from ICI therapy (13–15). Therefore, development of novel biomarkers in the hope of better prediction of the response to ICI therapy are urgently required.

Several biomarkers have been developed for predicting the benefit of ICI therapy for melanoma patients, including PD-L1 expression (16), tumor mutation burden (TMB) (17), interferon-γ signal (18, 19), and tumor infiltrating lymphocytes (TILs) (20). However, the immunohistochemistry analysis of PD-L1 varies significantly among different antibodies (21), thereby making it difficult to define the positive threshold of PD-L1 expression. In addition, the whole-genome sequences from 183 melanoma samples revealed that the burden of mutations is more frequent in cutaneous compared with acral and mucosal melanoma (22). Thus, the widespread detection value of TMB in acral and mucosal subtypes are limited.

The past decade has witnessed rapid progress in tumor genomics. Some studies utilized RNA sequencing data to establish immune-related gene signatures for the evaluation of immune response and prognosis in melanoma (23, 24). Unfortunately, none has been confirmed to be translated into clinical application owing to the small size of discovery data and lack of sufficient validation (25). Nowadays, a series of immunotherapy data regarding PD-1/PD-L1 or CTLA-4 blockade in melanoma patients have been reported all over the world. Integrated analyses may provide a complete picture of ICI therapy in different populations and summarize more superior predictive biomarkers. However, the information of gene expression profiling (GEP) was measured using different sequence platforms, which is not applied to normalizing gene expression levels through traditional approaches (26). Furthermore, the potential biological heterogeneity across datasets was also a challenge. Recently one method based on the construction of immune-related gene pairs (IRGPs) from GEP can be an excellent choice, which calculates the relative ranking of gene expression levels without the requirement for data preprocessing and has been demonstrated to establish robust models for the application of cancer classification (27–29). Hence, it is imperative to identify novel biomarkers based on IRGPs for guiding ICI therapy.

In this study, we integrated three published datasets of metastatic melanoma treated with anti-PD-1 (n = 120) and constructed an immune-related gene pair index (IRGPI). The IRGPI was constructed based on 31 immune-related gene pairs (IRGPs) consisting of 51 immune-related genes (IRGs), which may be a promising biomarker for predicting the response of ICI therapy and survival outcomes in melanoma patients. The predictive performance of IRGPI was also validated in Peking University Cancer Hospital (PUCH, n = 55) and VanAllen15 (n = 42) datasets treated with PD-1 or CTLA-4 blockade. Furthermore, the analyses of the TME, the immune cell infiltration, and biological functions of different IRGPI groups were also performed, which demonstrated that IRGPI may be an indicator of the immune status of the TME.

## Materials and Methods

### Patients and GEP

From March 2016 to March 2019, 55 melanoma patients treated with anti-PD-1 therapy were recruited for this study from PUCH. Formalin-fixed, paraffin-embedded pretreatment tumor samples were obtained from all patients. We separated all the clinical and pathological data by medical record review, including sex, age, primary site, metastasis status, and clinical efficacy. Tumor responses were evaluated using the Response Evaluation Criteria in Solid Tumors (RECIST) version 1.1, including complete response (CR), partial response (PR), stable disease (SD), and progressive disease (PD). CR and PR were regarded as responders, while PD and SD were regarded as non-responders. In this study, overall survival (OS) and progression-free survival (PFS) were used as the primary and secondary survival endpoints, respectively. Gene expression data for the PUCH cohort was based on the Illumina NovaSeq 6000 platform. The details of processing the GEP of the PUCH cohort have been described in our previous study (30). This study was conducted according to the Declaration of Helsinki Principles and approved by the Medical Ethics Committee of PUCH. Informed consent for the use of material in medical research was obtained from all participants.

### External Data Acquisition

We obtained RNA-seq and clinical data from four publicly available cohorts of melanoma patients treated with anti-PD-1 or anti-CTLA-4 therapy, including Gide19 (n = 41) (31), Hugo16 (n = 28) (32), Riaz17 (n = 51) (33), and VanAllen15 (n = 42) (34). Data of Gide19 cohort (PRJEB23709) were downloaded from the European Nucleotide Archive database (ENA; https://www.ebi.ac.uk/ena). Data of Hugo16 cohort (GSE91061) and Riaz17 cohort (GSE78220) were downloaded from the Gene Expression Omnibus database (GEO; https://www.ncbi.nlm.nih.gov/geo/). Data of VanAllen15 cohort (phs000452.v2.p1) were downloaded from the database of Genotypes and Phenotypes (dbGap; http://www.ncbi.nlm.nih.gov/gap). The treatment response to immunotherapy consisted of CR/PR/SD/PD according to RECIST 1.1 guidelines, which were used in our analysis.

Moreover, we downloaded the RNA-sequencing data of all available cutaneous melanoma samples from The Cancer Genome Atlas (TCGA) database through the GDC tool (http://portal.gdc.cancer.gov/). The survival data of these patients were extracted from cbioPortal (http://www.cbioportal.org). Patients with OS less than one month were excluded from our analysis. In addition, we separated the TMB data of melanoma patients in the TCGA-SKCM cohort from The Cancer Immunome Atlas (http://tcia.at/home) (35).

### Construction of the IRGPI

We constructed a predictive signature based on IRGs gathered from the ImmPort Web portal (https://www.immport.org/home) (36). Two IRGs constituted one IRGP and formed as “IRG-A|IRG-B”. The score of IRGPI was generated through pairwise comparison of gene expression levels in specific samples. When the expression level of IRG-A was higher than IRG-B, the IRGP was assigned a score of 1; otherwise, the IRGP score was 0. IRGPs with score of 0 or 1 in over 80% of the specimens were regarded as IRGPs with constant values, which does not contribute to the difference of patient survival (37). Therefore, we excluded these IRGPs with constant values from our analysis.

The Gide19, Hugo16, and Riaz17 cohorts merged into the meta cohort, which was used for the construction of IRGPI. Firstly, we used the log-rank test to investigate the correlation of each IRGP to patients’ OS in the meta cohort. According to the analysis results, IRGPs with a false discovery rate (FDR) < 0.001 were candidates to build the IRGPI. Then, the multivariate Cox regression analyses were performed to obtain the hub IRGPs and the respective coefficients. Finally, the IRGPI formula was defined as follows:


$$\text{IRGPI}=\sum_{\text{i}=1}^{\text{n}}\text{score}\;\text{of}\;\text{IRG}{\text{P}}_{\text{i}}\;*\text{coeffecien}{\text{t}}_{\text{i}}$$

### Validation of the IRGPI

The predictive and prognostic values of IRGPI for immunotherapy were validated in PUCH and VanAllen15 cohorts. Based on the treatment response to immunotherapy, we conducted the receiver operating characteristic (ROC) curve analysis to estimate the prediction accuracy of IRGPI. Using the cut-off value that generated the maximum Youden index (38), the patients were divided into IRGPI-high and IRGPI-low groups. Then, the log-rank tests were conducted for comparison of the survival outcomes between two IRGPI groups.

### Tumor Immune Microenvironment Analysis

The transcriptomic data of TCGA-SKCM (skin cutaneous melanoma) cohort were used for analyzing the association of IRGPI with immune-related features. Using the IRGPI formula, we calculated the IRGPI score of each patient in the TCGA-SKCM cohort. The cut-off value for the IRGPI was determined on the basis of the association with patients’ OS by using X-tile software (version 3.6.1) (39). Based on two bioinformatic analyses of GEP data in the TCGA-SKCM cohort, we calculated the enrichment of immune cells between two IRGPI groups. Briefly, we used Estimation of STromal and Immune cells in MAlignant Tumor tissues using Expression data (ESTIMATE) method to calculate the immune score and ESTIMATE score of patients (40). CIBERSORT was further used to distinguish 22 immune cells, such as T cell types, B cell types, NK cells, and myeloid cell types (41). In the signaling analysis, we conducted gene set enrichment analysis (GSEA) to distinguish which immune-related pathways were markedly different between IRGPI-high and IRGPI-low groups.

To further characterize the tumor immune microenvironment between two IRGPI groups, we performed single simple GSEA on some previously published immune-related signatures (19, 42–45) and compared the score between IRGPI-high and IRGPI-low groups.

### Statistical Analysis

All statistical analyses were performed using the R software (version 3.6.3) and Prism 8. Survival analyses were performed using the R packages “survival” and “survminer”. The signature of IRGPs was obtained using the R package “glmnet”. Univariate and multivariate Cox regression analyses were conducted using the R package “survival”. ROC curve analyses were performed using the R package “survivalROC”. ESTIMATE analysis was conducted using the R package “estimate”. CIBERSORT analysis was processed using the R packages “e1701”, “preprocessCore”, and “limma”. All statistical analyses were two-sided, and *P* < 0.05 was considered as statistically significant.

## Results

### Patients Characteristics

**Figure 1:**
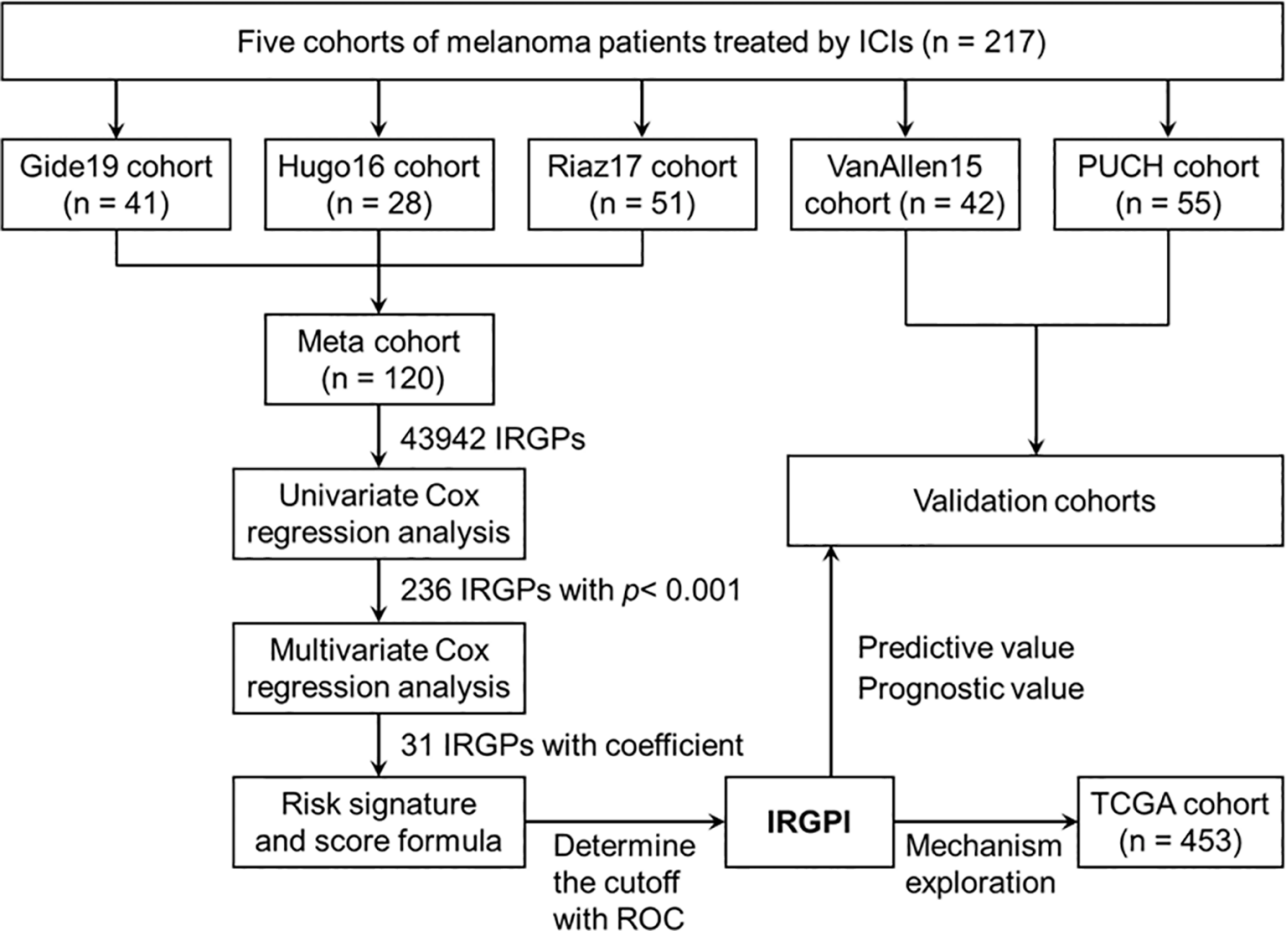
The flowchart showing the scheme of this study.

The flowchart of this study design is presented in **Figure 1**. A total of 217 patients treated with ICI from five cohorts were included in this study. We constructed the IRGPI based on the meta cohort (n = 120), which consisted Gide19 (n = 41), Hugo16 (n = 28), and Riaz17 (n = 51) cohorts. The PUCH (n = 55) and VanAllen15 (n = 42) cohorts were used for validation the prediction model. The clinicopathological characteristics are summarized in **Table 1**. The median follow-up is 13.1~32.7 months in five cohorts. Notably, 43.6% of patients in PUCH cohort were acral melanomas, which have been reported to be the main subtype of melanoma in Asians. However, the vast majority of patients in other cohorts were cutaneous melanoma.

**Table 1:** Clinicopathological characteristics of five immunotherapy cohorts included in this study.

Patient characteristics	Training cohorts			Validation cohorts	
	Gide19	Hugo16	Riaz17	VanAllen15	PUCH
No. of patients	41	28	51	42	55
Median age in yrs (range)	66 (37-90)	61 (19-84)	–	61 (22-83)	51 (27-72)
Sex, n (%)					
Male	26 (63.4)	20 (71.4)	–	28 (66.7)	17 (30.9)
Female	15 (36.6)	8 (28.6)	–	14 (33.3)	38 (69.1)
Primary site, n (%)					
Acral	–	–	1 (2.0)	–	24 (43.6)
Mucosal	–	3 (10.7)	7 (13.7)	2 (4.8)	8 (14.5)
Cutaneous	–	21 (75.0)	32 (62.7)	37 (88.1)	18 (32.7)
Ocular		–	4 (7.9)	3 (7.1)	–
Unknown	–	4 (14.3)	7 (13.7)	–	5 (9.1)
Metastasis status, n (%)					
M0	–	1 (3.6)	1 (1.9)	1 (2.4)	10 (18.2)
M1a	–	2 (7.1)	11 (21.6)	3 (7.1)	16 (29.1)
M1b	–	3 (10.7)	8 (15.7)	7 (16.7)	18 (32.7)
M1c	–	22 (78.6)	23 (45.1)	31 (73.8)	11 (20.0)
Unknown	–	–	8 (15.7)	–	–
BRAF V600, n (%)	–	12 (42.9)	14 (27.4)	–	–
Prior MAPKi, n (%)	–	12 (42.9)	–	4 (9.5)	–
Treatment, n (%)					
Anti-PD-1	41 (100)	28 (100)	51 (100)	–	55 (100)
Anti-CTLA-4	–	–	–	42 (100)	
Best overall response, n (%)					
CR	4 (9.8)	5 (17.9)	3 (5.9)	–	1 (1.8)
PR	15 (36.6)	10 (35.7)	7 (13.7)	–	13 (23.6)
CR/PR	–	–		19 (45.2)	
SD	6 (14.6)	–	16 (31.4)	–	6 (10.9)
PD	16 (39.0)	13 (46.4)	25 (49.0)	23 (54.8)	35 (63.6)
Median PFS (months)	9.0	–	–	2.8	3.9
Median OS (months)	29.3	32.7	21.1	13.1	28.1

MAPKi, MAPK pathway inhibitors; Anti-PD-1, anti-programmed death-1; Anti-CTLA-4, anti-cytotoxic T lymphocyte antigen-4; CR, complete response; PR, partial response; SD, stable disease; PD, progressive disease; PFS, progression-free survival; OS, overall survival.

### Construction and Definition of the IRGPI

Among the 2487 IRGs from the ImmPort database, 1138 IRGs commonly occurred in the GEP of five cohorts and 1295044 IRGPs were calculated. We excluded 1251102 IRGPs (96.6%) with constant values in any cohort and 43942 IRGPs remained for subsequent construction of the IRGPI (**Table S1**). According to the univariate Cox regression analyses of the correlation between each IRGP and patients’ OS in the meta cohort, 236 IRGPs with adjusted *P* < 0.001 were selected as prognostic IRGPs (**Table S2**). Then, we performed the multivariate Cox regression analyses to filtrate IRGPs to construct the IRGPI. Finally, 31 IRGPs were filtrated to define the IRGPI (**Table 2** and **Figure S1A**). The IRGPI consisted of 51 unique IRGs, most of which encoded molecules involved in antimicrobials, cytokines, and cytokine receptors.

**Table 2:** Model information of IRGPI.

IRG-A	Full name	Immune pathway	IRG-B	Full name	Immune pathway	Coefficient
*CD1B*	CD1b molecule	Antigen Processing and Presentation	AMHR2	anti-Mullerian hormone receptor type 2	Cytokine Receptors, TGFb Family Member Receptor	-0.133719837
*CD1C*	CD1c molecule	Antigen Processing and Presentation	GDNF	glial cell derived neurotrophic factor	Cytokines, TGFb Family Member	-0.006407801
*CD1E*	CD1e molecule	Antigen Processing and Presentation	NGF	nerve growth factor	Cytokines	-0.118650926
*HLA-C*	major histocompatibility complex, class I, C	Antigen Processing and Presentation, NaturalKiller Cell Cytotoxicity	SPP1	secreted phosphoprotein 1	Cytokines	-0.000146032
*HSPA6*	heat shock protein family A (Hsp70) member 6	Antigen Processing and Presentation	PI15	peptidase inhibitor 15	Antimicrobials	-0.006568346
*IFNG*	interferon gamma	Antigen Processing and Presentation, Antimicrobials, Cytokines, Interferons, NaturalKiller Cell Cytotoxicity, TCR Signaling Pathway	NTS	neurotensin	Cytokines	-0.208747404
*RELB*	RELB proto-oncogene, NF-kB subunit	Antigen Processing and Presentation	NFATC4	nuclear factor of activated T cells 4	BCR Signaling Pathway, NaturalKiller Cell Cytotoxicity, TCR Signaling Pathway	-0.001379582
*CXCL13*	C-X-C motif chemokine ligand 13	Antimicrobials, Chemokines, Cytokines	PLAU	plasminogen activator, urokinase	Antimicrobials, Chemokines, Cytokines	-0.042607265
*XCL1*	X-C motif chemokine ligand 1	Antimicrobials, Chemokines, Cytokines	FABP6	fatty acid binding protein 6	Antimicrobials	-0.179318494
*SFTPD*	surfactant protein D	Antimicrobials	CR2	complement C3d receptor 2	BCR Signaling Pathway	0.248588976
*MMP9*	matrix metallopeptidase 9	Antimicrobials	NOX4	NADPH oxidase 4	Antimicrobials	-0.126050935
*RBP7*	retinol binding protein 7	Antimicrobials	PRF1	perforin 1	NaturalKiller Cell Cytotoxicity	0.040708869
*IFIH1*	interferon induced with helicase C domain 1	Antimicrobials	VAV3	vav guanine nucleotide exchange factor 3	BCR Signaling Pathway, NaturalKiller Cell Cytotoxicity, TCR Signaling Pathway	-0.601441422
*IDO1*	indoleamine 2,3-dioxygenase 1	Antimicrobials	CD72	CD72 molecule	BCR Signaling Pathway	-0.238320598
*IDO1*	indoleamine 2,3-dioxygenase 1	Antimicrobials	SECTM1	secreted and transmembrane 1	Cytokines	-0.16744787
*IRF1*	interferon regulatory factor 1	Antimicrobials	HMOX1	heme oxygenase 1	Antimicrobials	-0.00086748
*IRF1*	interferon regulatory factor 1	Antimicrobials	IL1R1	interleukin 1 receptor type 1	Cytokine Receptors, Interleukins Receptor	-0.115025852
*ZYX*	zyxin	Antimicrobials	IRF9	interferon regulatory factor 9	Antimicrobials	0.117305566
*TNFAIP3*	TNF alpha induced protein 3	Antimicrobials	IL1R1	interleukin 1 receptor type 1	Cytokine Receptors, Interleukins Receptor	-0.209151382
*HMOX1*	heme oxygenase 1	Antimicrobials	IL32	interleukin 32	Cytokines	0.199502086
*CCR7*	C-C motif chemokine receptor 7	Antimicrobials, Chemokine Receptors, Cytokine Receptors	IL11	interleukin 11	Cytokines, Interleukins	-0.036970847
*PTGDR*	prostaglandin D2 receptor	Antimicrobials, Cytokine Receptors	EGF	epidermal growth factor	Cytokines	-0.088054831
*RAC3*	Rac family small GTPase 3	BCR Signaling Pathway, NaturalKiller Cell Cytotoxicity	NR1D1	nuclear receptor subfamily 1 group D member 1	Cytokine Receptors	0.174898131
*CD19*	CD19 molecule	BCR Signaling Pathway	EGF	epidermal growth factor	Cytokines	-0.012058023
*INPP5D*	inositol polyphosphate-5-phosphatase D	BCR Signaling Pathway	IL1R1	interleukin 1 receptor type 1	Cytokine Receptors, Interleukins Receptor	-0.014592558
*CXCR3*	C-X-C motif chemokine receptor 3	Chemokine Receptors, Cytokine Receptors	IL11	interleukin 11	Cytokines/Interleukins	-0.12999067
*EGF*	epidermal growth factor	Cytokines	TNFRSF11A	TNF receptor superfamily member 11a	Cytokine Receptors, TNF Family Members Receptors	0.254354052
*IL33*	interleukin 33	Cytokines, Interleukins	RARG	retinoic acid receptor gamma	Cytokine Receptors	-0.27380975
*IL7*	interleukin 7	Cytokines, Interleukins	PRF1	perforin 1	NaturalKiller Cell Cytotoxicity	0.062199575
*IL20RB*	interleukin 20 receptor subunit beta	Cytokine Receptors, Interleukins Receptor	TNFRSF10C	TNF receptor superfamily member 10c	Cytokine Receptors, NaturalKiller Cell Cytotoxicity, TNF Family Members Receptors	0.049115859
*TEK*	TEK receptor tyrosine kinase	Cytokine Receptors	CD28	CD28 molecule	TCR Signaling Pathway	0.046180545

### Evaluation of the Prediction Accuracy of IRGPI for the Efficacy of ICI Therapy

**Figure 2:**
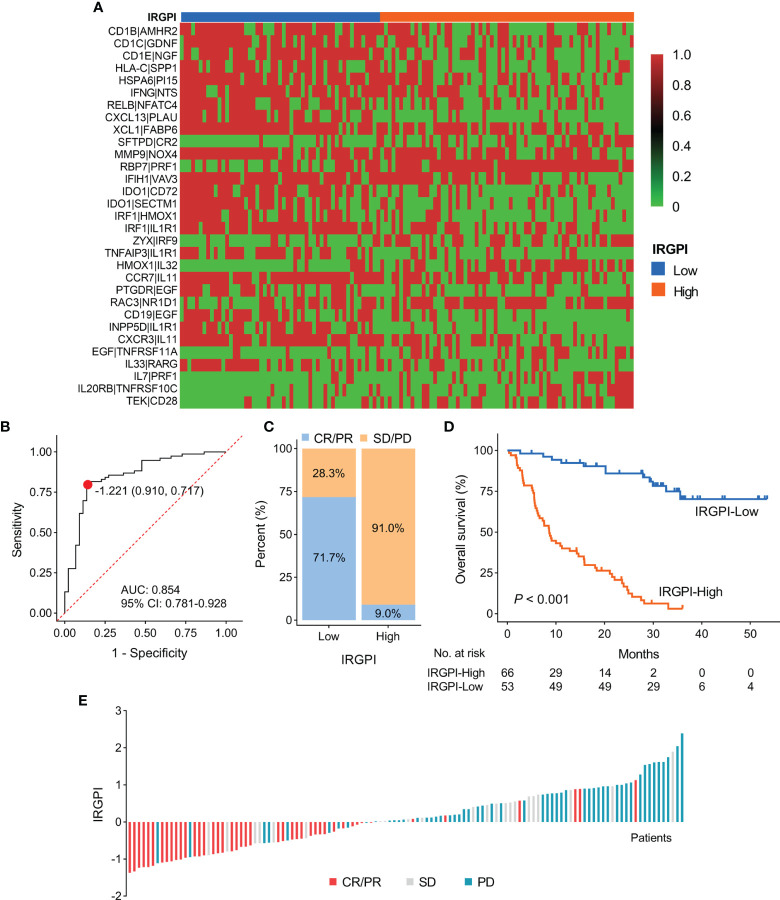
Construction and evaluation of IRGPI in the discovery cohort. **(A)** A heatmap of the identified 31 IRGPs with corresponding IRGPI groups. **(B)** ROC curve for the predictive performance of IRGPI. **(C)** The rate of durable clinical response for patients with high and low IRGPI scores. **(D)** Kaplan-Meier plots of overall survival segregated by IRGPI score with cut-off points selected according to the Youden index. **(E)** Waterfall plot of IRGPI for distinct clinical response groups. IRGPI, immune-related gene pair index; IRGPs, immune-related gene pairs; ROC, receiver operating characteristic; AUC, area under curve; CI, confidence interval.

Based on the IRGPI formula, we calculated each patient’s IRGPI score in the meta cohort and exhibited the result in a heatmap (**Figure 2A**). We conducted ROC curve analysis to evaluate the prediction accuracy of IRGPI for the efficacy of ICI therapy in the meta cohort. With a cut-off value based on the Youden index of -1.221, we found that IRGPI-low group patients were correctly classified as CR/PR with a sensitivity of 71.7% (38/53). Further, IRGPI-high group patients were successfully classified as SD/PD with a specificity of 91.0% (61/67). The overall accuracy of IRGPI was 82.5% (99/120) with AUC of 0.854 (**Figure 2B**). IRGPI-low group showed a higher ORR than IRGPI-high group (71.7% vs. 9.0%; **Figures 2C, E**). Moreover, we evaluated the relationship between the IRGPI score and OS/PFS in the meta cohort. Kaplan-Meier survival analysis revealed that IRGPI-low group patients had significantly longer OS (*P* < 0.001; **Figure 2D**). The median PFS for IRGPI-high group patients was markedly shorter than IRGPI-low group patients in the Gide19 cohort (*P* < 0.001; **Figure S2A**). The pooled hazard ratio (HR) along with 95% confidence interval (CI) for the association between high IRGPI score and OS in 119 cases of patients was 8.02 (3.91~15.19), and no significant heterogeneity among the three datasets was observed (I^2^ = 0%, *P* = 0.98, **Figure S1B**). Overall, the IRGPI showed a superior prediction for the benefit of ICI therapy in the meta cohort.

### Validation of the Robustness of IRGPI in Predicting the Efficacy of ICI Therapy

**Figure 3:**
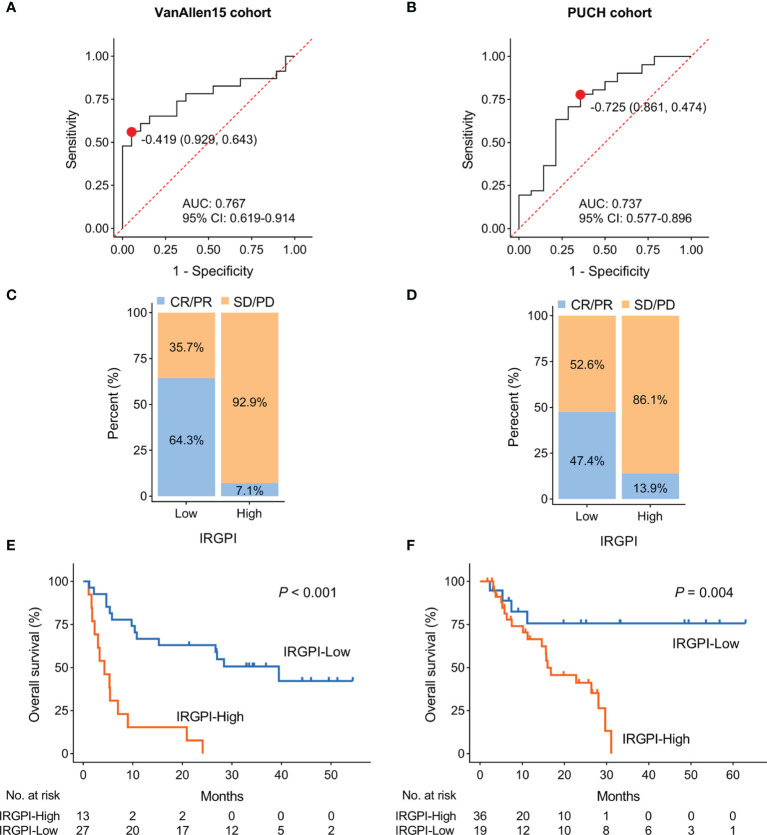
Validation the performance of IRGPI in two cohorts. **(A, B)** ROC curves for the predictive performance of IRGPI in VanAllen15 and PUCH cohorts, respectively. **(C, D)** The rate of durable clinical response for patients with high and low IRGPI scores in VanAllen15 and PUCH cohorts, respectively. **(E, F)** Kaplan-Meier plots of overall survival segregated by IRGPI score with cut-off points selected according to the Youden index in VanAllen15 and PUCH cohorts, respectively. IRGPI, immune-related gene pair index; ROC, receiver operating characteristic; AUC, area under curve; CI, confidence interval.

To verify the robustness of IRGPI in predicting the efficacy of ICI therapy, we assessed the correlation of IRGPI score with overall response rate and survival outcomes in VanAllen15 and PUCH cohorts. In the VanAllen15 cohort, the IRGPI successfully identified 31 of 42 patients with an overall accuracy of 73.8% and an AUC of 0.767 (**Figure 3A**). Similarly, in the PUCH cohort, the IRGPI demonstrated an overall accuracy of 72.7% (40/55) and AUC of 0.737 (**Figure 3B**). The ORR of IRGPI-high group was lower than IRGPI-low group in VanAllen15 cohort (64.3% vs. 7.1%; **Figures 3C** and **S3A**) and PUCH cohort (47.4% vs. 13.9%; **Figures 3D** and **S3B**), respectively. As expected, higher IRGPI conferred poor survival outcomes in VanAllen15 cohort (OS: *P* < 0.001, PFS: *P* < 0.001; **Figures 3E** and **S2B**) and PUCH cohort (OS: *P* = 0.004, PFS: *P* = 0.015; **Figures 3F** and **S2C**), respectively. These results confirmed that the IRGPI is reliable for the prediction of ICI therapy responsiveness in VanAllen15 and PUCH cohorts.

### Association of IRGPI With Tumor Immune Microenvironment in Melanoma

**Figure 4:**
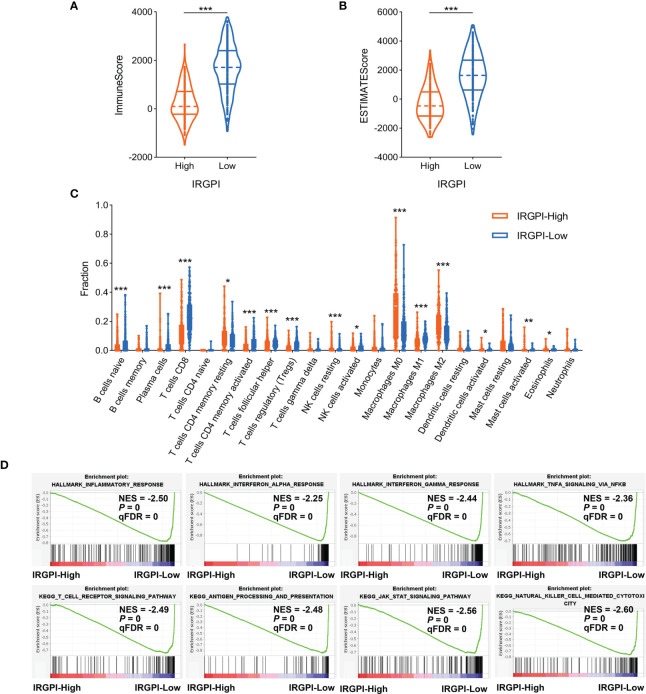
Comparison of immune microenvironment characteristics according to IRGPI status. **(A, B)** ESTIMATE algorithm revealed the ImmuneScore and ESTIMATEScore between IRGPI-high and IRGPI-low groups. **(C)** Evaluation of 22 immune cell infiltrating using the CIBERSORT method. **(D)** GSEA plots of immune-related pathways in comparison between IRGPI-high and IRGPI-low groups. IRGPI, immune-related gene pair index; GSEA, gene set enrichment analysis. *P < 0.05, **P < 0.01, ***P < 0.001.

Reportedly, the infiltration of immune cells, especially CD8^+^ T cells, is associated with immunotherapy response in many types of cancer (46). Based on the above results, we further investigated the relationship between IRGPI and tumor immune microenvironment features in melanoma patients. The TCGA-SKCM cohort was stratified into IRGPI-high and IRGPI-low groups using X-tile software (**Table S3** and **Figure S4**). Firstly, we calculated the immune score and ESTIMATE score of patients in TCGA-SKCM cohort by ESTIMATE algorithm. The data showed that both the immune score and ESTIMATE score were considerably increased in IRGPI-low group compared with IRGPI-high group (**Figures 4A, B**). Secondly, the CIBERSORT analytical tool was used to estimate the proportions of 22 types of immune cells in each SKCM sample. The results revealed that the infiltration levels of CD8^+^ T cells, activated memory CD4^+^ T cells, naive B cells, and NK cells in IRGPI-high group were lower than that in IRGPI-low group, while resting memory CD4^+^ T cells, M0 and M2 macrophages showed the opposite trend (**Figure 4C**). Finally, we performed GSEA to identify which pathways were enriched at specific IRGPI levels. As shown in **Figure 4D**, the pathways of inflammatory response, interferon response, antigen processing and presentation, and T cell receptor signaling were markedly upregulated in IRGPI-low group.

### Correlation of IRGPI to Other Potential Immunotherapy Biomarkers in Melanoma

**Figure 5:**
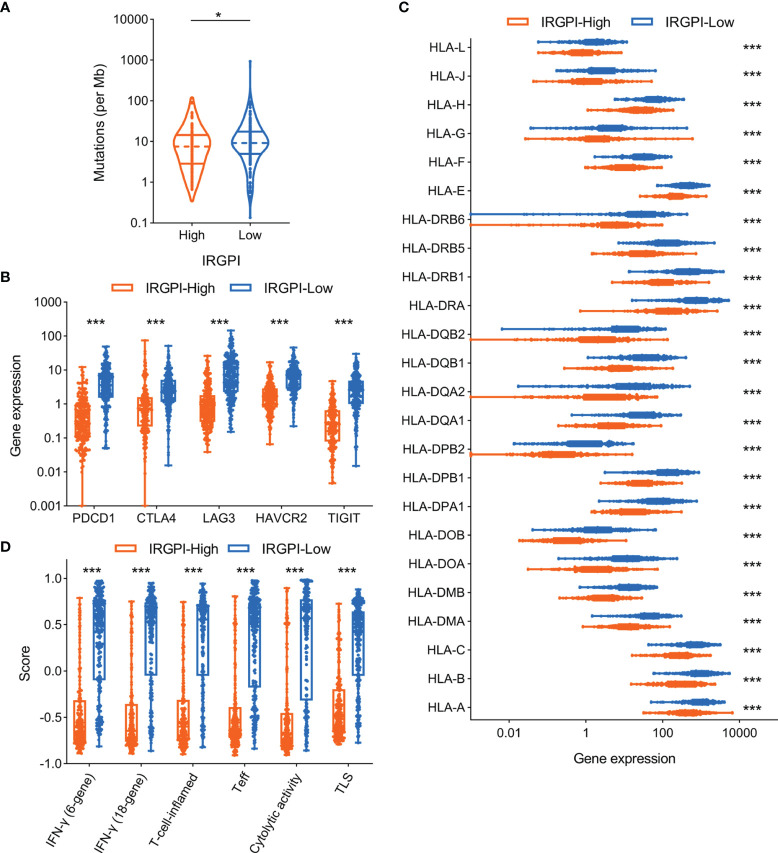
Association of IRGPI to other potential biomarkers in melanoma. **(A)** Comparison of tumor mutation burden level according to IRGPI status. **(B)** Correlation of IRGPI to immune inhibitory receptors, including PDCD1, CTLA4, LAG3, HAVCR2, TIGIT. **(C)** The profile of HLA member expression levels between IRGPI-high and IRGPI-low groups. **(D)** Box plot of the immune-related signatures in comparison of the IRGPI-high and IRGPI-low groups. IRGPI, immune-related gene pair index; HLA, human leukocyte antigen; IFN, interferon; Teff, effective T cells; TLS, tertiary lymphoid structure. *P < 0.05, ***P < 0.001.

A series of potential biomarkers have been developed to predict the response of ICI therapy in malignant tumors, such as TMB, immune inhibitory receptor expression levels. We analyzed the relationship between IRGPI and TMB in TCGA-SKCM cohort and the results showed that higher IRGPI conferred lower TMB (**Figure 5A**). As expected, the expression levels of immune inhibitory receptors (including PD-1, CTLA-4, LAG3, TIM-3, and TIGIT) showed the same trend as TMB between IRGPI-high and IRGPI-low groups (**Figure 5B**). Moreover, the deficiency of human leukocyte antigen (HLA) could impair antigen presentation and initiate antitumor immunity, which consequently resulting in primary resistance to immunotherapy (47). We then investigated the correlation of IRGPI to the expression levels of HLA members and the data indicated most HLA members were substantially upregulated in IRGPI-low group compared with IRGPI-high group (**Figure 5C**).

Some immune-related GEP signatures have been described to predict the benefit of ICI therapy in melanoma (**Table S4**). We therefore compared these GEP signature scores between IRGPI-high and IRGPI-low groups. Consistent with other biomarkers, these GEP signature scores were significantly downregulated in IRGPI-high group compared with IRGPI-low group (**Figure 5D**).

## Discussion

Over the past decades, the incidence of malignant melanoma has continued to increase, but the mortality has decreased, largely due to the rapid development of ICI and targeted therapies (48). Compared with the excellent clinical efficacy of ICI therapy in melanoma, the investigations of its biomarkers are relatively insufficient. The data from the real world revealed that durable responses and favorable long-term outcomes are limited a proportion of melanoma (12). Thus, more attention should be paid to the discovery and establishment of novel biomarkers for selecting patients who may benefit from ICI therapy.

In this study, we integrated the data of ICI therapy of melanoma patients from our center and other four Western cohorts (31–34), and constructed an individualized immune predictive signature (IRGPI). The rate of durable clinical response for IRGPI-low patients in the discovery cohort, VanAllen15 and PUCH cohorts were 71.7%, 64.3% and 47.4%, respectively. Further, the percentage of non-responder in IRGPI-high group in discovery cohort, VanAllen15 and PUCH cohorts were 91%, 92.9% and 86.1%, respectively. The AUCs of ROC curve were all more than 0.7 in the discovery and validation cohorts. This reflected the good prediction accuracy and sensitivity of IRGPI for ICI therapy. The patient classification based on IRGPI also showed significantly different survival outcomes. Meanwhile, it is noted that the patients from our center are mainly acral and mucosal subtypes, while the patients from four Western cohorts are mainly cutaneous melanomas, which is consistent with the previous studies (49, 50). Considering the differential subtypes of melanoma and robust prediction accuracy across five cohorts, we reasonably assume that IRGPI is a reliable biomarker for guiding ICI therapy in both Western and Eastern patients with melanoma.

The IRGPI consisted of 51 unique IRGs, of which 36 encoded molecules involved in antimicrobials, cytokines, and cytokine receptors, which play vital roles in the regulation of the response to tumor immune microenvironment. Meanwhile, many of these IRGs have been demonstrated to be correlated with PD-L1 signaling and anti-PD-1 therapy, such as *MMP9* (matrix metallopeptidase 9) and *EGF* (epidermal growth factor). Zhao et al. found that TGFβ pathway inhibition promoted the proliferation expansion of stromal fibroblasts, thereby facilitating MMP9-dependent cleavage of PD-L1 surface expression, leading to PD-1 blockade resistance in melanoma models (51). Furthermore, inhibition of MMP9 promoted the therapeutic efficacy of PD-1 blockade, with a marked reduction of tumor burden and extension of survival time (52). Li et al. discovered that the immunosuppressive activity of PD-L1 was tightly regulated by ubiquitination and N-glycosylation, in which glycogen synthase kinase 3β (GSK3β) could induce phosphorylation-dependent proteasome degradation of PD-L1 (53). In addition, EGF could stabilize PD-L1 *via* GSK3β inactivation in basal-like breast cancer (53). Therefore, blocking of EGF signaling using gefitinib resulted in the destabilization of PD-L1, enhancing antitumor T cell immunity and the treatment response of PD-1 blockade in syngeneic mouse models. What’s more, some IRGs (including *IFNG*, *PRF1*, *IDO1*, *CXCL13*) were included in an IFN-γ-related T cell-inflamed GEP, which have been developed into a clinical-grade assay for evaluating the treatment efficacy of pembrolizumab in pan-tumors (19). As expected, the GSEA results of our study showed that lower IRGPI conferred upregulated interferon response and inflammatory signaling. The above data and analyses indicated that IRGPI could predict T cell inflammation in melanoma and explain the relationship between IRGPI and ICI therapy responsiveness to some extent.

TILs, especially CD8^+^ T cells, can be used for predicting ICI therapy responsiveness and survival outcomes (46). In our study, we calculated the relative proportion of 22 types of immune cells based on the CIBERSORT algorithm and the results revealed that melanoma samples with high IRGPI harbored more infiltration of CD8+ T cells, activated memory CD4^+^ T cells, naive B cells, and NK cells, which further elucidated the reason why IRGPI-high patients with melanoma can benefit from ICI therapy. However, the infiltration levels of regulatory T cells (Tregs) in IRGPI-high patients were also significantly higher compared with that in IRGPI-low patients, which was contradictory with the previous report of Tregs with an immunosuppressive role in TME (54). Further investigations are required to evaluate the infiltration levels of Tregs using immunohistochemistry or flow cytometry.

There are some limitations, unresolved concerns, and potential perspectives in our study. First, the current study combined the data from different datasets, which can sometimes present a selection bias, due to various therapy settings, different pre-existing mutations, and baseline patient characteristics. Although this gene-pair based approach we used in this study does not require normalization of GEP, this bias across cohorts is inevitable. Second, for the IRGPI, there are still some genes whose function are not fully elucidated. Further studies, such as knockdown or overexpression of IRGs in melanoma cell lines, are required to verify the role of these genes. Moreover, the basic experiments were also lacking to examine the immune cell infiltrating and PD-L1 expression of patients treated with ICI therapy. Finally, the patients in PUCH cohort were treated with different anti-PD-1 antibodies from various pharmaceutical companies, which may lead to drug bias. Compared with two previous studies (NCT02821000 and NCT02836795) of PD-1 blockade for treating melanoma patients, the data showed the ORR of two types of anti-PD-1 antibodies in mucosal subtype were 13.3% and 0, respectively (14, 55). Thus, further studies, preferably in a prospective setting, are required to stringently evaluate the correlation of IRGPI to the immunotherapy response and survival outcomes.

## Conclusions

In summary, we constructed an individualized immune predictive signature (IRGPI), which could robustly predict the ICI therapy responsiveness and long-term survival outcomes. In addition, IRGPI may be an indicator of the immune characteristics of the TME in melanoma patients. These findings indicated that IRGPI might serve as a novel marker for treating of melanoma with ICI, which needs to be validated in prospective clinical trials.

## Data Availability Statement

The datasets presented in this study can be found in online repositories. The names of the repository/repositories and accession number(s) can be found below: https://ngdc.cncb.ac.cn/search/?dbId=&q=HRA000524.

## Ethics Statement

The studies involving human participants were reviewed and approved by Peking University Cancer Hospital & Institute. The patients/participants provided their written informed consent to participate in this study.

## Author Contributions

SC and YK were involved in conception and design of the study. JuY performed and evaluated the experiment. XW and JiY helped to analyze the results. YK and XW provided materials or patients. JuY wrote the manuscript. All authors contributed to the article and approved the submitted version.

## Funding

This work was supported by grants from National Natural Science Foundation of China (82002906, 81902789, 82002897) and CSCO-Roche Cancer Research Fund 2019 (Y-Roche2019/2-0028).

## Conflict of Interest

The authors declare that the research was conducted in the absence of any commercial or financial relationships that could be construed as a potential conflict of interest.

## Publisher’s Note

All claims expressed in this article are solely those of the authors and do not necessarily represent those of their affiliated organizations, or those of the publisher, the editors and the reviewers. Any product that may be evaluated in this article, or claim that may be made by its manufacturer, is not guaranteed or endorsed by the publisher.

## Supplementary Material

Supplementary Figure 1 Forest plot of different IRGPI groups. **(A)** Multivariate Cox analysis of 31 hub immune-related gene pairs. **(B)** Forest plot of high IRGPI score with poor OS in patients from three datasets.

Supplementary Figure 2 The performance of the IRGPI in predicting progression-free survival in three cohorts.

Supplementary Figure 3 Waterfall plot of IRGPI for distinct clinical response groups in VanAllen15 and PUCH cohorts.

Supplementary Figure 4 X-tile plots of the IRGPI scores in TCGA-SKCM cohort.

## Abbreviations

AUC, Area Under Curve; CR, complete response; ESTIMATE, Estimation of STromal and Immune cells in MAlignant Tumor tissues using Expression data; FDR, false discovery rate; GEP, gene expression profiling; CI, confidence interval; GSEA, gene set enrichment analysis; HLA, human leukocyte antigen; HR, hazard ratio; ICI, immune checkpoint inhibitors; IRGs, immune-related genes; IRGPs, immune-related gene pairs; IRGPI, immune-related gene pair index; ORR, overall response rate; OS, overall survival; PD, progressive disease; PFS, progression-free survival; PUCH, Peking University Cancer Hospital; PR, partial response; RECIST, Response Evaluation Criteria in Solid Tumors; ROC, receiver operating characteristic SD, stable disease; TCGA, The Cancer Genome Atlas; TILs, tumor infiltrating lymphocytes; TME, tumor microenvironment; TMB, tumor mutation burden; Tregs, regulatory T cells.
