# Supplementary figures and images for: An Immune-Related Gene Pair Index Predicts Clinical Response and Survival Outcome of Immune Checkpoint Inhibitors in Melanoma
Source: Front Immunol. 2022 Feb 24;13:839901. doi: 10.3389/fimmu.2022.839901 (PMC8907429; doi:10.3389/fimmu.2022.839901)

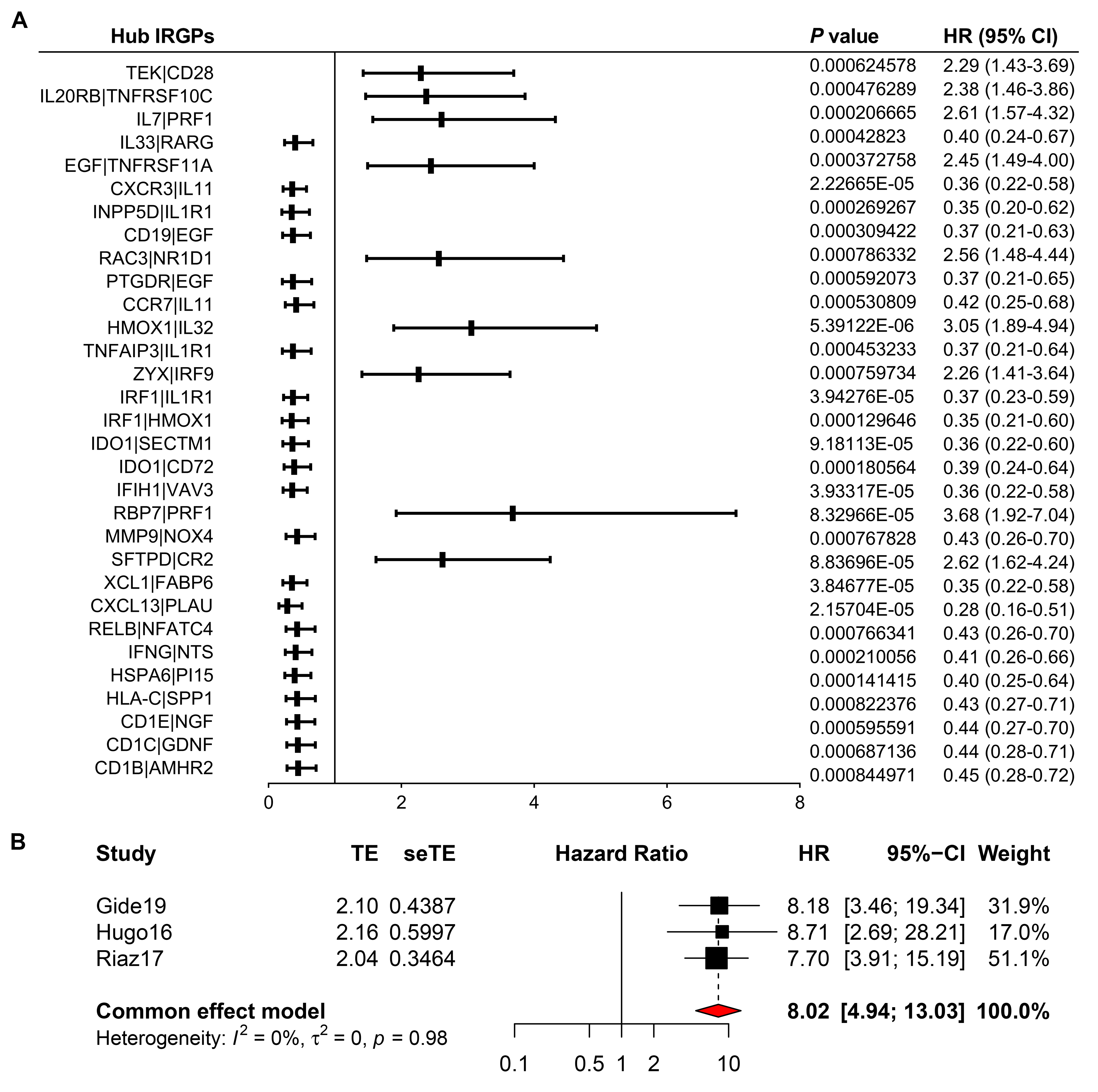

Supplement: Supplementary Figure 1 — Forest plot of different IRGPI groups. (A) Multivariate Cox analysis of 31 hub immune-related gene pairs. (B) Forest plot of high IRGPI score with poor OS in patients from three datasets. [file Image_1.tif]

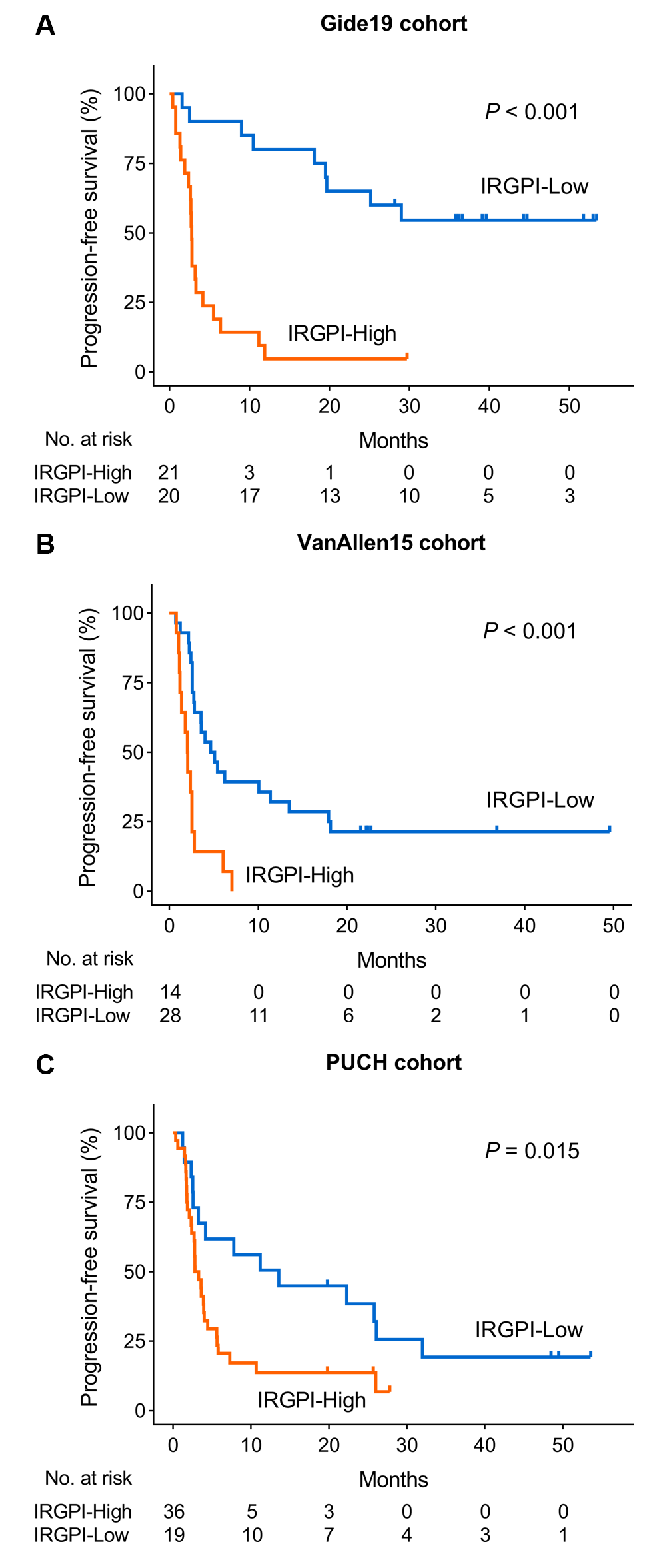

Supplement: Supplementary Figure 2 — The performance of the IRGPI in predicting progression-free survival in three cohorts. [file Image_2.tif]

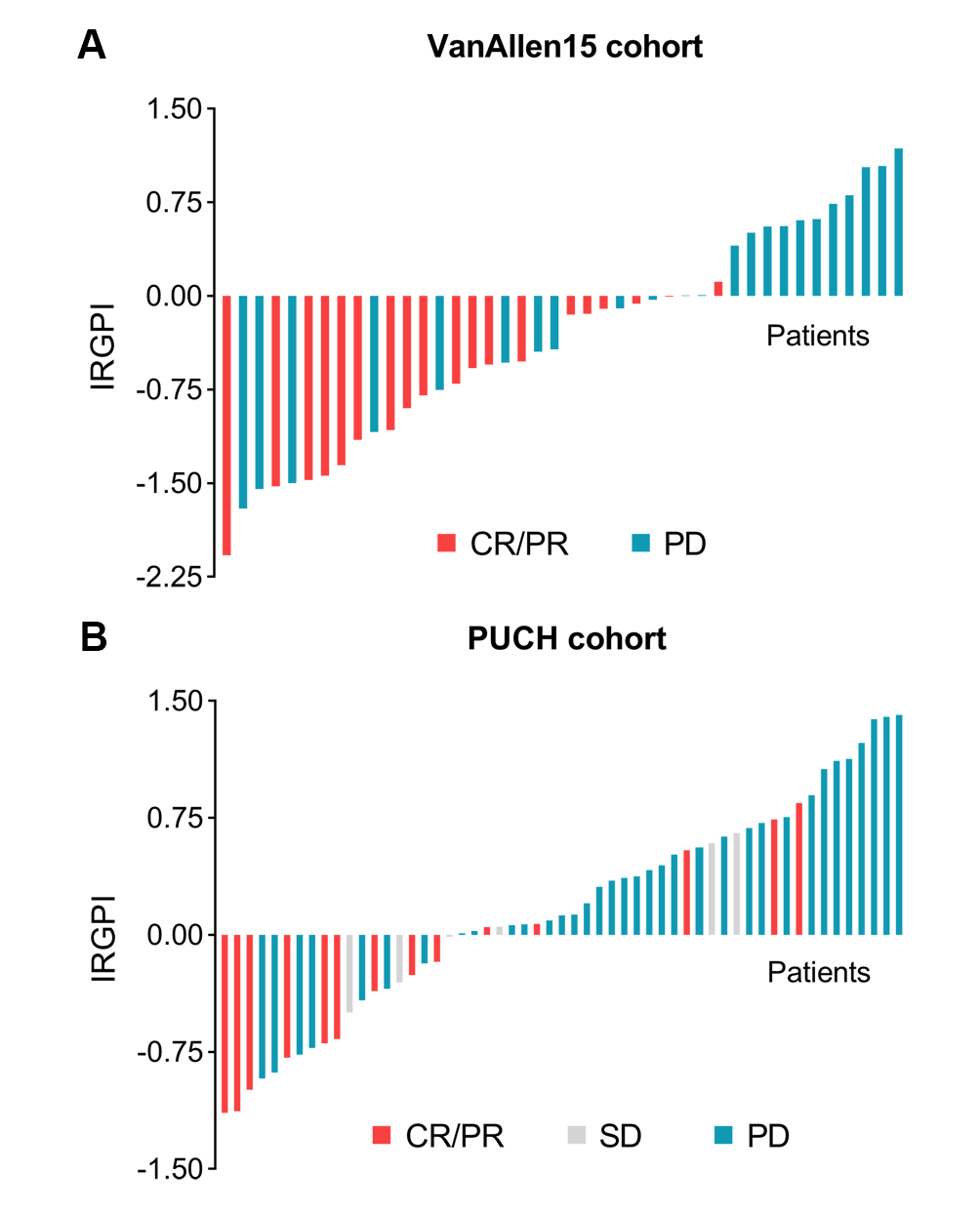

Supplement: Supplementary Figure 3 — Waterfall plot of IRGPI for distinct clinical response groups in VanAllen15 and PUCH cohorts. [file Image_3.tif]

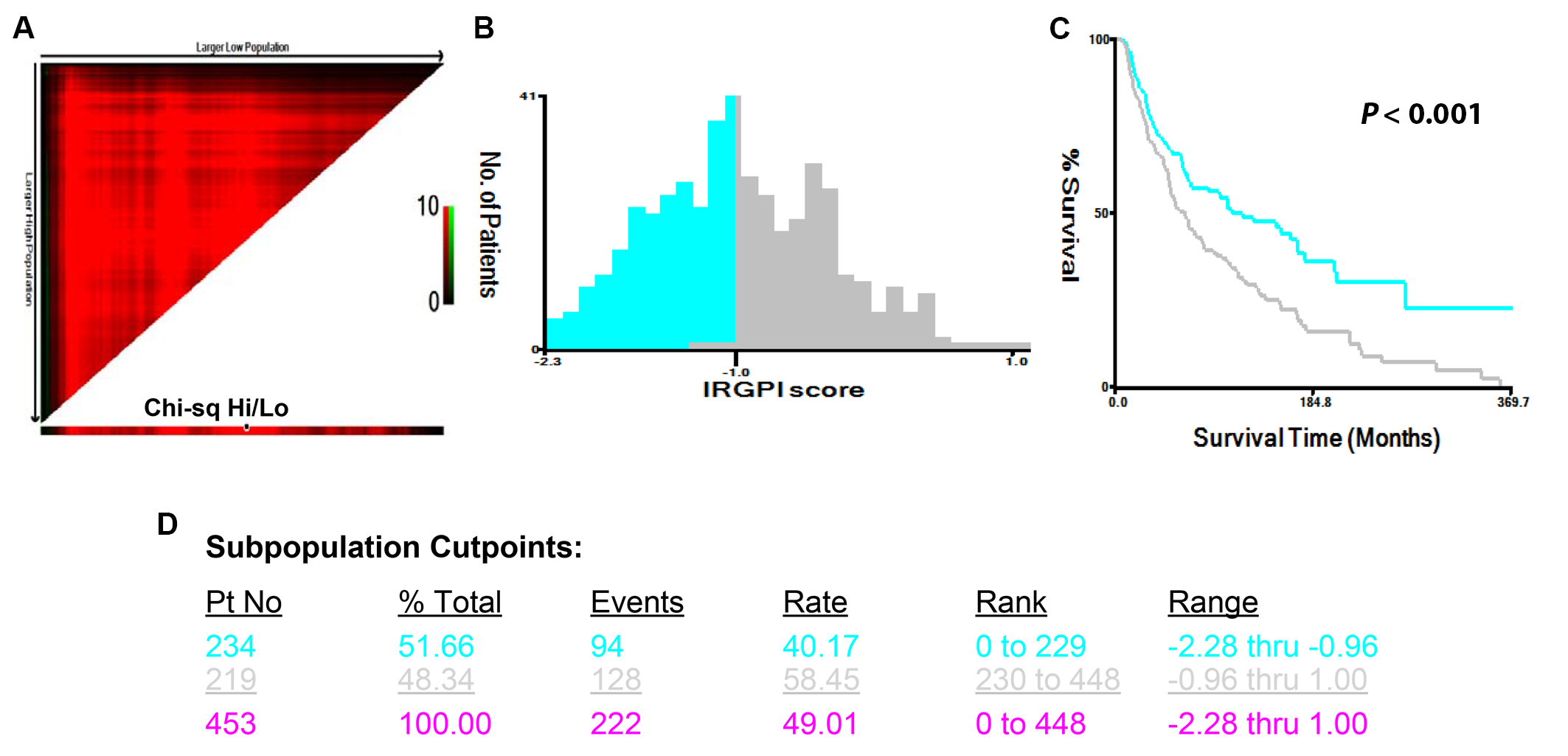

Supplement: Supplementary Figure 4 — X-tile plots of the IRGPI scores in TCGA-SKCM cohort. [file Image_4.tif]
